# PSAT1 enhances the efficacy of the prognosis estimation nomogram model in stage-based clear cell renal cell carcinoma

**DOI:** 10.1186/s12885-024-12183-z

**Published:** 2024-04-13

**Authors:** Jun Wang, Xiaoming He, Yuanyuan Mi, Yong Q. Chen, Jie Li, Rong Wang

**Affiliations:** 1 Department of Urology, First Affiliated Hospital of Nanjing Medical University, Nanjing Medical University, Nanjing, 210008 China; 2 Department of Urology, Affiliated Hospital of Jiangnan University, Jiangnan University, Wuxi, 214122 China; 3 Wuxi Maternal and Child Health Hospital, Wuxi School of Medicine, Jiangnan University, Jiangsu, 214002 China; 4 Wuxi School of Medicine, Jiangnan University, Wuxi, 214122 China

**Keywords:** Clear cell renal cell carcinoma, PSAT1, Prognosis, Nomogram model

## Abstract

**Background:**

Clear cell renal cell carcinoma (ccRCC) is associated with a high prevalence of cancer-related deaths. The survival rates of patients are significantly lower in late-stage ccRCC than in early-stage ccRCC, due to the spread and metastasis of late-stage ccRCC, surgery has not reached the goal of radical cure, and the effect of traditional radiotherapy and chemotherapy is poor. Thus, it is crucial to accurately assess the prognosis and provide personalized treatment at an early stage in ccRCC. This study aims to develop an efficient nomogram model for stratifying and predicting the survival of ccRCC patients based on tumor stage.

**Methods:**

We first analyzed the microarray expression data of ccRCC patients from the Gene Expression Omnibus (GEO) database and categorized them into two groups based on the disease stage (early and late stage). Subsequently, the GEO2R tool was applied to screen out the genes that were highly expressed in all GEO datasets. Finally, the clinicopathological data of the two patient groups were obtained from The Cancer Genome Atlas (TCGA) database, and the differences were compared between groups. Survival analysis was performed to evaluate the prognostic value of candidate genes (PSAT1, PRAME, and KDELR3) in ccRCC patients. Based on the screened gene PSAT1 and clinical parameters that were significantly associated with patient prognosis, we established a new nomogram model, which was further optimized to a single clinical variable-based model. The expression level of PSAT1 in ccRCC tissues was further verified by qRT-PCR, Western blotting, and immunohistochemical analysis.

**Results:**

The datasets GSE73731, GSE89563, and GSE150404 identified a total of 22, 89, and 120 over-expressed differentially expressed genes (DEGs), respectively. Among these profiles, there were three genes that appeared in all three datasets based on different stage groups. The overall survival (OS) of late-stage patients was significantly shorter than that of early-stage patients. Among the three candidate genes (PSAT1, PRAME, and KDELR3), PSAT1 was shown to be associated with the OS of patients with late-stage ccRCC. Multivariate Cox regression analysis showed that age, tumor grade, neoadjuvant therapy, and PSAT1 level were significantly associated with patient prognosis. The concordance indices were 0.758 and 0.725 for the 3-year and 5-year OS, respectively. The new model demonstrated superior discrimination and calibration compared with the single clinical variable model. The enhancer PSAT1 used in the new model was shown to be significantly overexpressed in tissues from patients with late-stage ccRCC, as demonstrated by the mRNA level, protein level, and pathological evaluation.

**Conclusion:**

The new prognostic prediction nomogram model of PSAT1 and clinicopathological variables combined was thus established, which may provide a new direction for individualized treatment for different-stage ccRCC patients.

**Supplementary Information:**

The online version contains supplementary material available at 10.1186/s12885-024-12183-z.

## Introduction

Clear cell renal cell carcinoma (ccRCC) is a major histological subtype of renal cell carcinoma (RCC), and accounts for approximately 60%-85% of RCC cases. ccRCC is characterized by epithelial cells of renal proximal convoluted tubules [1, 2]. The early stages of ccRCC typically present as asymptomatic, with approximately 25%-30% patients exhibiting metastasis at the time of diagnosis [3]. The relapse or distant metastasis rate for ccRCC patients after radical nephrectomy exceeds 20%. Furthermore, the resistance of ccRCC to radiotherapy and chemotherapy results in a poor prognosis [4, 5]. Thus, improved prognosis prediction of advanced ccRCC patients will greatly assist clinicians in decision-making. Moreover, the identification of key genetic drivers for progression can aid in the development of new treatments.

Relevant prognostic factors have been observed in addition to the American Joint Committee on Cancer (AJCC) Tumor Node Metastasis (TNM) stage [6]. It is worth noting that gene expression profiling has the potential to classify different tumor types because of the significant involvement of genes in tumor development and metastasis [7]. The rapid development of gene sequencing technology has made Gene Expression Omnibus (GEO) and The Cancer Genome Atlas (TCGA) databases increasingly important in bio-informatics analysis [8, 9]. These databases offer sequencing data for discovering new functional genes and analyzing their impact on prognosis. Thus, the analysis of the objective need for clinical variable gene combinations and nomograms can serve as an effective tool in the development of individualized patient treatment strategies.

Nomograms are established based on Cox regression analysis results and are widely used for cancer prognosis, primarily because of their ability to reduce statistical predictive models to a single numerical estimate of the probability of an event, such as death or recurrence [10, 11]. In ccRCC patients, a nomogram combining differentiation-related gene (DRG)-based risk score and prognostic clinicopathological variables was previously constructed to provide a visual method for determining prognosis [12]. However, another nomogram based on risk gene signature and clinical features may provide a practical method for recurrence prediction and facilitate personalized management of ccRCC patients after surgery [13]. Thus, research on the prediction model based on tumor stage requires further investigation.

This study found the key prognostic genes affecting different stages of ccRCC. Phosphoserine aminotransferase 1 (PSAT1) is a protease of class V pyridoxal phosphate-dependent aminotransferase family. A gene mutation in PSAT1 leads to metabolic and genetic disorders such as phosphoserine aminotransferase deficiency, serine deficiency, and Neu-Laxova syndrome, wherein patients require postnatal serine and glycine supplementation for symptom alleviation. In recent years, an increasing number of investigations have shown that PSAT1 is highly associated with the occurrence, development, treatment, and prognosis of various cancers [14–18]. Therefore, the objective of this study was to conduct a comprehensive bioinformatics analysis to identify the prognostic genes in patients at different stages of ccRCC and to develop a new nomogram model for predicting overall survival (OS) in patients with late-stage ccRCC based on the data from the GEO and TCGA databases.

## Materials and methods

### Acquisition of microarray data

The discovery phase involved the identification of datasets comparing mRNA expression in tissues of patients with late-stage (stage III+ stage IV) ccRCC with that in the tissues of patients with early-stage (stage I+ stage II) ccRCC. Gene expression profiles of GSE73731 (with 256 samples), GSE89563 (with 16 samples), and GSE150404 (with 60 samples) were obtained from the National Center for Biotechnology Information (NCBI) GEO database (https://www.ncbi.nlm.nih.gov/geo/). The GSE73731 dataset was based on the GPL570 platform, whereas GSE89563 and GSE150404 were based on the GPL17692 platform.

### Screening for integrated differentially expressed genes (DEGs) at different stages

The GEO2R tool, which relies on the R package “Limma” provided by the GEO database, was used for identifying DEGs in each dataset. The cut-off criteria for screening over-expressed DEGs were adjusted *p*-values < 0.05 and log2FC > 1. The significantly up-regulated genes were separately extracted.

Genes over-expressed in all datasets were identified by constructing a Venn diagram using an online tool (http://bioinformatics.psb.ugent.be/webtools/Venn/), which depicted three lists of up-regulated genes. The expression levels of all genes and survival analysis of selected genes at different stages were verified using the Assistant for Clinical Bioinformatics (ACBI) tool (https://www.aclbi.com). The levels of potential hub genes were determined using R software to create a heatmap.

### Collection of clinical and bioinformatics data

The TCGA database was accessed on June 9, 2023, and the clinical data including tumors RNA expression data of 532 ccRCC patients were collected (https://tcga-data.nci.nih.gov/). Clinical parameters included sex, age, race, pathologic T stage, pathologic N stage, pathologic M stage, grade, neoadjuvant therapy, vital status, and follow-up duration (days). Depending on the stage of ccRCC patients, we divided the patients into late-stage and early-stage groups with the help of the ACBI module of TCGA (https://www.aclbi.com/static/index.html#/tcga). The RNA sequencing expression profiles and corresponding clinical information by stage-group downloaded from the TCGA dataset (https://portal.gdc.com) were matched with the TCGA-ccRCC dataset (https://tcga-data.nci.nih.gov/). Considering the influence of surgical factors, we excluded the data of patients whose follow-up time was less than 30 days. The median RNA expression value in the two groups was regarded as the cut-off to the RNA expression levels as high or low in each group.

### Development of risk prediction model

According to the TCGA data, we developed a nomogram combining gene expression with clinical information (new model) for the prediction of 3-year and 5-year OS in individuals with different stages of ccRCC. Another nomogram only using clinical variables was developed for a head-to-head comparison with the first comprehensive model in ccRCC patients at different stages.

### Patients and tissue specimens

A total of 20 pairs of ccRCC specimens were obtained from patients who underwent radical nephrectomy or partial nephrectomy at the Affiliated Hospital of Jiangnan University. None of the patients in our study received neoadjuvant chemotherapy. In all, 20 matched fresh ccRCC specimens (10 pairs of late-stage cases and 10 pairs of early-stage cases) and adjacent noncancerous renal tissues were selectively used for qRT-PCR, Western blotting, and immunohistochemical analysis. The diagnosis for each patient was confirmed by histopathological analysis. Informed consent was obtained from the patients before inclusion in the study, and the study protocol was approved by the Ethics Committee of the Affiliated Hospital of Jiangnan University.

### RNA extraction and qRT-PCR assays

Total RNA was extracted by RNA-easy (RC101, Vazyme, Nanjing, CN) according to the reagent instructions. In all, 1 µg of total RNA was used for cDNA synthesis using a cDNA reverse transcription kit (R323, Vazyme, Nanjing, CN). Real-time PCR was performed in triplicates on a Bio-Rad CFX96 PCR system to detect PSAT1expression. The results were normalized to the expression of GAPDH. The primer sequences are listed below: PSAT1-F: ACAGGAGCTTGGTCAGCTAAG, PSAT1-R: CATGCACCGTCTCATTTGCG; GAPDH-F: GGAGCGAGATCCCTCCAAAAT, GAPDH-R: GGCTGTTGTCATACTTCTCATGG.

### Immunohistochemistry analysis

The ccRCC tissue samples were obtained from the Affiliated Hospital of Jiangnan University according to institutional guidelines. Tissue paraffin blocks were sectioned, and stained with antibodies specific to PSAT1 (10501-1-AP: 1:400, Proteintech, Wuhan, CN), followed by scanning with a Pannoramic Scanner (3DHISTECH, Budapest, Hungary).

### Western blotting

The kidney tissues were treated destructed and then lysed by boiling for 10 min in sample buffer (2% SDS, 10% glycerol, 10% β-mercaptoethanol, bromophenol blue, and Tris-HCl, pH = 6.8). The lysates were fractionated by SDS-PAGE and the isolates were transferred to PVDF membranes (Millipore, IPVH00010, NH, US). The blots were probed with specific primary antibodies followed by a secondary antibody and the membranes were then detected by ECL (Sigma, WBULS0500, MO, US). PSAT1 (10501-1-AP: 1:10000) and GAPDH (66009-1-Ig; 1:10000) antibodies were purchased from Proteintech Group (IL, US). Secondary antibodies were conjugated with HRP (Proteintech Group; SA00001-1, SA00001-2; 1:10000). Uncropped WB are shown in Fig. S3.

### Statistical analyses

Statistical analyses were conducted using SPSS version 27.0 (SPSS Inc., IBM Corp., Armonk, NY, USA) and R software for Windows, version 4.2.3. Data are presented as mean ± SD or median and range. Student’s t test was performed for normally distributed continuous variables, while Mann-Whitney U test was performed for non-normally distributed data. Chi square or Fisher’s exact test was applied to compare categorical variables.

The Cox proportional hazard regression model was used to estimate the hazard ratio and its 95% confidence interval (CI) for each potential risk factor, and data were visualized through Forest plots. The stepwise multivariate Cox regression analysis included inclusion and exclusion criteria of type I error = 0.1.

Discrimination reflects the ability of a model to distinguish events and non-events correctly, and these were validated using C-statistics. The Concordance index (C-index) is analogous to the area under the receiver operating characteristic (ROC) curve. The predictive capacity of models was summarized using ROC curves [19]. Calibration refers to the closeness between the predicted probabilities and the actual outcomes, and this was validated using calibration plots [20].

A two-sided *p*-value of < 0.05 was considered statistically significant.

## Results

### Identification of DEGs in ccRCC patients at different stages

**Fig. 1:**
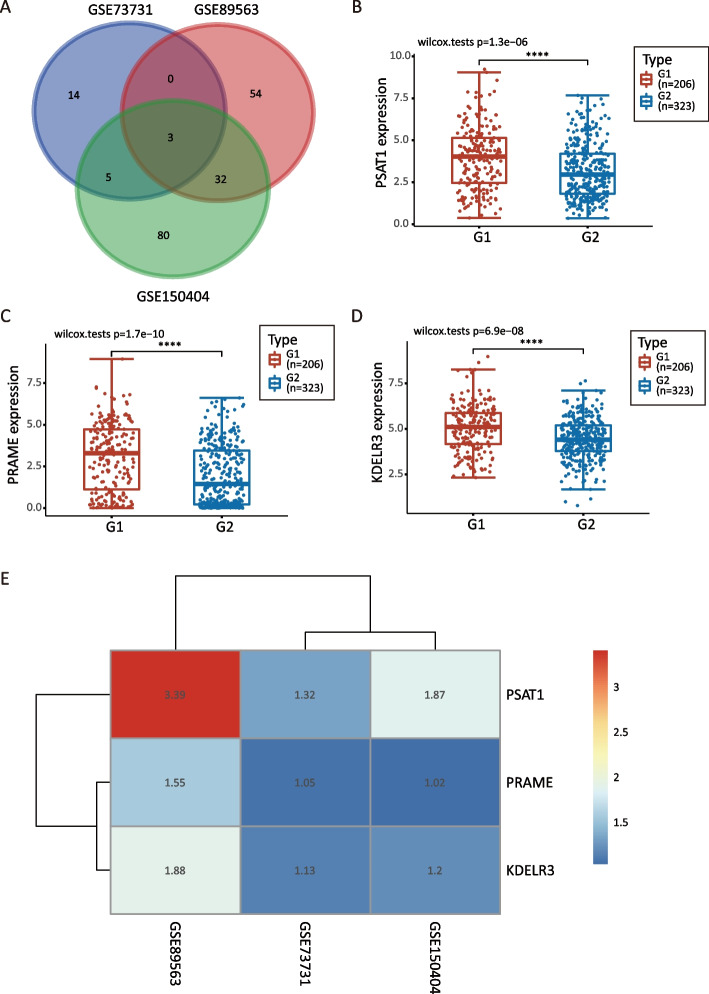
Screening and identification of differentially co-expressed genes. **A** Different colors represent the number of up-regulated genes in different datasets, and the middle intersection represents the number of co-expressed genes in the three GSE (GSE73731, GSE89563, GSE150404) datasets. **B** PSAT1 expression. **C** PRAME expression. **D** KDELR3 expression. Red dots represent the number of cases in the late-stage ccRCC group, blue dots represent the number of cases in the early-stage ccRCC group. Horizontal line in the middle of the box: represents the median. Top and bottom lines of the box: represent the upper and lower quartiles. The bin size represents the quartile spacing. G1: late-stage group; G2: early-stage group. ****p < 0.0001. **E** The heatmap illustrates the expression levels of potential 3 genes. Each row represents the expression level of each gene in different samples, and each column represents the expression level of all genes in each sample

We downloaded three ccRCC gene expression profiles (GSE73731, GSE89563, and GSE150404) from the GEO database and screened 22, 89, and 120 over-expressed DEGs, respectively, using the GEO2R tool to differentiate between late-stage and early-stage patients. A Venn diagram was constructed (Fig. 1A) and three genes (PSAT1, PRAME, and KDELR3) that were over-expressed in the three profiles were identified. The three selected genes were verified in TCGA (Fig. 1B-D), and the gene PSAT1 was the most significantly differentially expressed (Fig. 1E).

### Determination of the effect of PSAT1 on the prognosis of late-stage patients

The clinicopathological data were integrated with the expression levels of PSAT1, PRAME, and KDELR3, which matched the corresponding data in the two databases based on row-name, and finally, we included 529 cases. The median of hub-gene expression level was used to divide patients into the up-regulated and down-regulated subgroups within different stage groups. Patients with a follow-up duration of < 30 days or those who had no clinical OS information were excluded. Finally, data of 499 ccRCC patients for OS in different disease stages was obtained, wherein the early-stage group included 303 patients and late-stage group included 196 patients.

Detailed characteristics were compared between the two groups. The mean ages of patients in the early-stage and late-stage groups were 59.58 ± 12.69 and 61.59 ± 11.40 years, respectively, which showed no statistically significant difference (*p* = 0.073). Besides, no significant difference was also observed between the two groups with regard to gender, ethnicity, and neoadjuvant therapy (all *p* > 0.05). However, a significant difference in the expressions of PSAT1, PRAME, KDELR3 was observed between groups; these genes were highly expressed in patients with late-stage ccRCC than in those with early-stage ccRCC (all *p* < 0.05) (Table 1).

**Table 1:** Comparison of relative factors between the two groups

Variables	Early stage	Late stage	t/χ2	*P* value
Age (years)	59.58 ± 12.69	61.59 ± 11.40	1.798	0.073
Gender			0.429	0.513
Male	197 (65.0)	133 (67.9)		
Female	106 (35.0)	63 (32.1)		
Ethnic			4.599	0.100
White	264 (87.1)	182 (92.9)	0.036	0.850
Asian	5 (1.7)	3 (1.5)	0.596	0.440
Black	34 (11.2)	11 (5.6)	4.587	0.032
Grade			108.968	< 0.001*
G1	12 (4.0)	0 (0.0)	2.793	0.131
G2	174 (57.4)	41 (20.9)	37.089	< 0.001*
G3	104 (34.3)	93 (47.5)	27.863	< 0.001*
G4	13 (4.3)	62 (31.6)	34.522	< 0.001*
G1 + G2	186 (61.4)	41 (20.9)	78.60	< 0.001*
G3 + G4	137 (38.6)	155 (79.1)		
Neoadjuvant			0.117	0.732
Yes	11 (3.1)	6 (3.6)		
No	292 (96.9)	190 (96.4)		
PSAT1 Expression			23.065	< 0.001*
High	125 (41.3)	124 (63.3)		
Low	178 (58.7)	72 (36.7)		
PRAME Expression			40.711	< 0.001*
High	117 (38.6)	133 (67.9)		
Low	186 (61.4)	63 (32.1)		
KDELR3 Expression			29.854	< 0.001*
High	122 (40.3)	128 (65.3)		
Low	181 (59.7)	68 (34.7)		

^*^Statistically significant *P* < 0.05

**Fig. 2:**
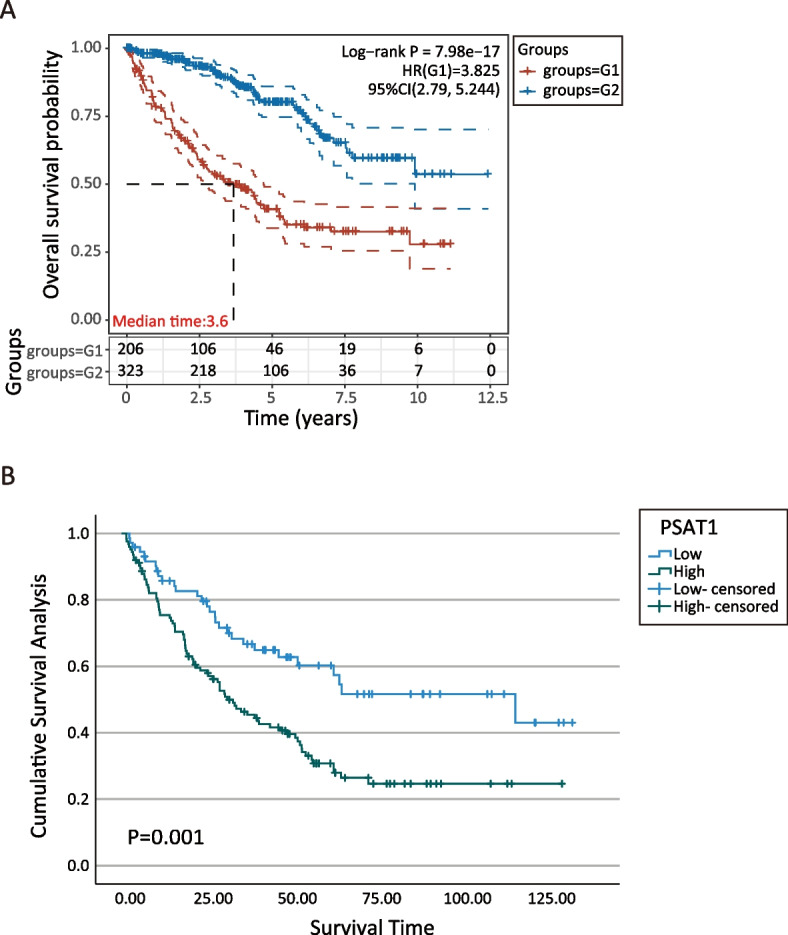
Kaplan-Meier plots for ccRCC patients. **A** Overall survival in different stage groups. The mortality rates of the two groups show a downward trend, which decreased rapidly at the beginning and then leveled off. The red curve represents the late-stage group, blue curve represents the early-stage group. **B** PSAT1 expression level about late-stage patients for overall survival. The mortality rates of the different expression level showed a downward trend, which decreased rapidly at the beginning and then leveled off. The blue curve represents the high-expression, green curve represents the low-expression. Follow-up time for horizontal axis and vertical axis for survival rate. Survival curves were obtained by linking the corresponding survival rates at each time point. G1: late-stage group; G2: early-stage group. *p* < 0.05 was considered statistically significant

Based on the ACBI-TCGA database, we found significance differences in the OS curves between the two groups (Fig. 2A, log-rank *p* < 0.001). Kaplan–Meier curves of OS according to gene expression level for hub-genes in the different stage groups showed that only PSAT1 expression exhibited statistical significance in terms of OS curves (Fig. 2B, log-rank *p* = 0.001). High levels of PSAT1 expression were associated with a poor prognosis in late-stage patients, whereas the other two genes did not have a prognostic value with regard to OS in patients with different stages of ccRCC (Fig. S1).

### Confirm risk/protect factors of prognosis

**Fig. 3:**
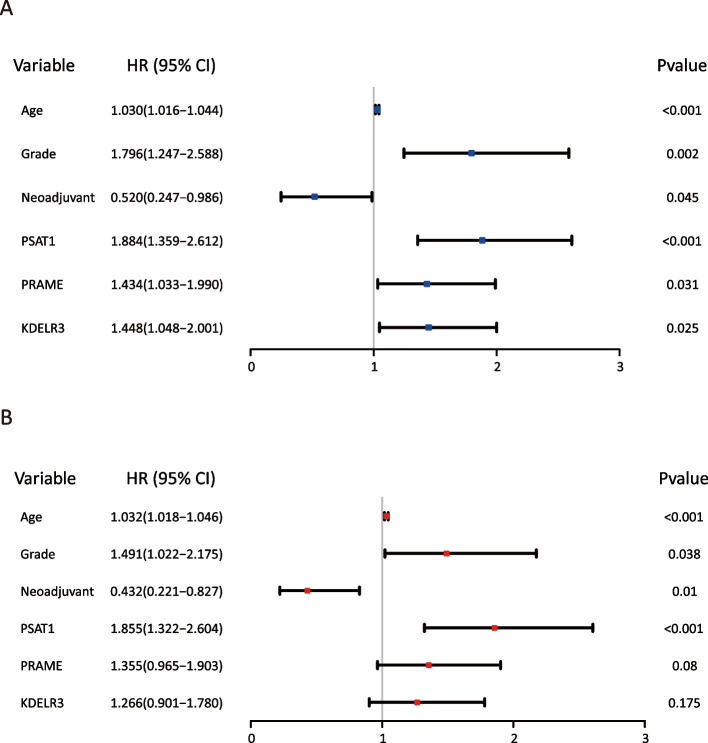
Influence factors for overall survival by Cox-regression analysis. **A** Univariate analysis. **B** Multivariate analysis. Short line parallel to the X-axis, the length of the line segment corresponds to the 95% CI. Abscissa values correspond on both ends of the line around 95% CI of two Numbers, the square line corresponding to the HR. Perpendicular to the X axis of the straight line, known as invalid. Crossing this line indicates that the results are not statistically significant. HR > 1 is on the right side of the line, indicating a risk factor. HR < 1 is on the left side of the line, indicating a protective factor. HR: Hazard Ratio; CI: Confidence internal. Statistically significant *p* < 0.05

To search for potentially relevant risk factors, univariate Cox-regression analysis revealed that six variables, age (*p* < 0.001), grade (*p* = 0.002), neoadjuvant therapy (*p* = 0.045), PSAT1 expression (*p* < 0.001), PRAME expression (*p* = 0.031), and KDELR3 expression (*p* = 0.025), showed a significant correlation with OS in the two stage groups of patients. The remaining parameters did not indicate significant statistical associations (all *p* > 0.05) (Fig. 3A and Table 2).

**Table 2:** COX regression analysis factors affecting overall survival

Variable	Univariate analysis			Multivariate analysis		
	HR	95%CI	*P*	HR	95%CI	*P*
Age (years)	1.030	1.016–1.044	< 0.001*	1.032	1.018–1.046	< 0.001*
Gender						
Male /Female	1.096	0.800-1.501	0.570			
Ethnic						
White /Asian /Black	1.019	0.750-1.386	0.903			
Grade						
G4 + G3/G2 + G1	1.796	1.247-2.588	0.002	1.491	1.022-2.175	0.038
Neoadjuvant						
Yes /No	0.520	0.247-0.986	0.045	0.432	0.221-0.827	0.010
PSAT1 Expression						
High/Low	1.884	1.359-2.612	<0.001*	1.855	1.322-2.604	<0.001*
PRAME Expression						
High/Low	1.434	1.033-1.990	0.031	1.355	0.965-1.903	0.080
KDELR3Expression						
High/Low	1.448	1.048-2.001	0.025	1.266	0.901-1.780	0.175

*HR* Hazard Ratio, *CI* Confidence internal, *G4* + *G3* High Grade (HG), *G2* + *G1* Low Grade (LG)

^*^Statistically significant *P* < 0.05

The significant risk factors determined in the univariate analysis were further evaluated via multivariate Cox-analysis. Finally, age [HR: 1.032, 95% CI: 1.018 to 1.046, *p* < 0.001], grade (HR: 1.491, 95% CI: 1.022 to 2.175, *p* = 0.038), and PSAT1 expression (HR: 1.855, 95% CI: 1.322 to 2.604, *p* < 0.001) were identified as independent risk factors of OS in the two groups, whereas neoadjuvant therapy (HR: 0.432, 95% CI: 0.221 to 0.827, *p* = 0.01) was identified as a protective factor (Fig. 3B and Table 2).

### Development of a nomogram combining gene and clinical variables

**Fig. 4:**
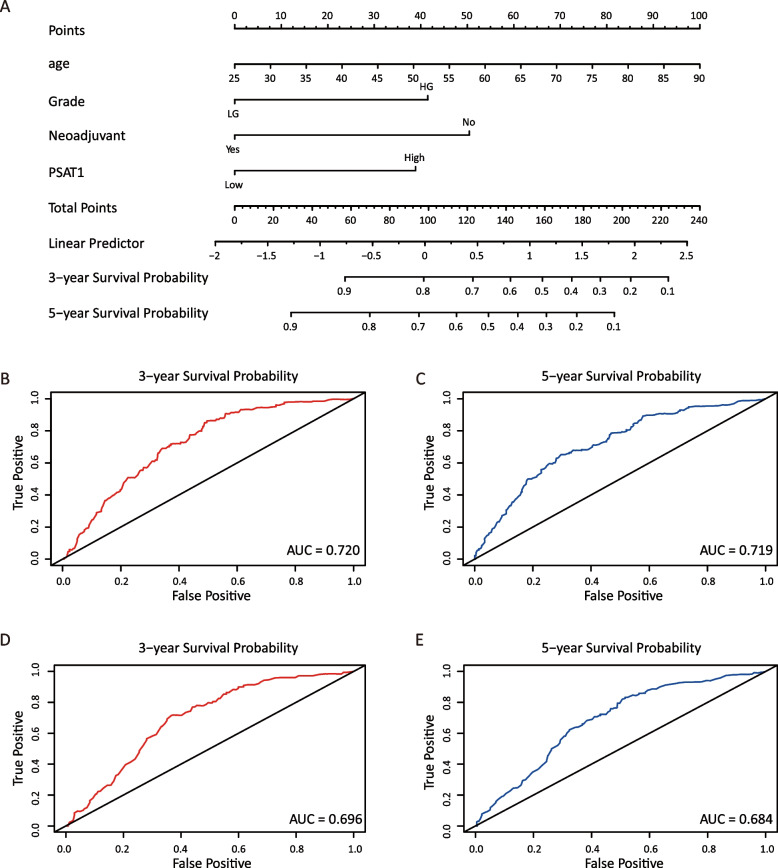
Predicting and verification of 3-year and 5-year overall survival. **A** The risk factors were represented by points on the axis, with each factor corresponding to a line drawn upward. The total points located on the axis indicated the probability of 3-year and 5-year overall survival, represented by a line drawn downward to the survival axis. **B** 3-year overall survival in new mode. **C** 5-year overall survival in new model. **D** 3-year overall survival in only clinical variables model. **E** 5-year overall survival in only clinical variables model. AUC represents the area under the ROC curve. AUC value is between 0.5 and 1, the closer to 1, the better the performance of model diagnosis, the higher the accuracy. (1) 0.7 < AUC < 0.9, indicating that the accuracy of the model is good and has certain clinical application value. (2) 0.5 < AUC < 0.7, indicating that the accuracy of this index/model is low and has little clinical application value. AUC: area under curve

Based on the abovementioned results, we developed a prediction model and generated a graphical nomogram predicting the probability of 3-year and 5-year OS in the late-stage compared with early-stage groups (Fig. 4A). PSAT1 was included in the nomogram of OS and the predictive accuracy of the nomogram calculated by AUC was 0.720 for 3-year OS and 0.719 for 5-year OS (Fig. 4B-C), which revealed moderate discriminatory ability.

**Fig. 5:**
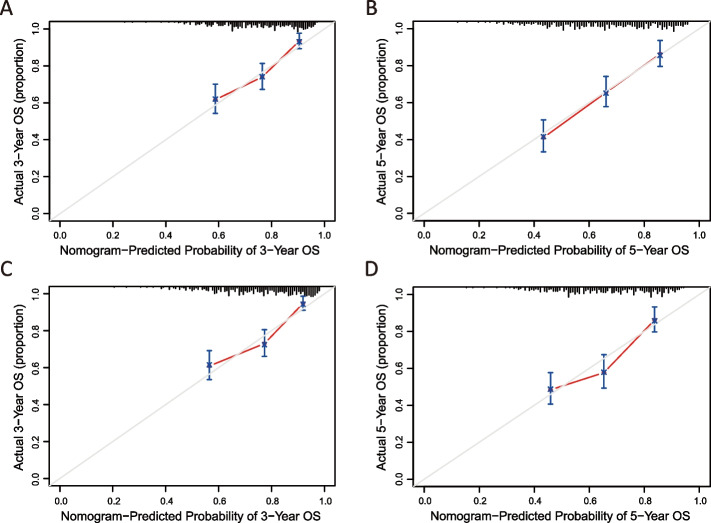
The calibration curve’s accuracy represents different models’ predictions. **A** 3-year overall survival in new model. **B** 5-year overall survival in new model. **C** 3-year overall survival based solely on clinical variables model. **D** 5-year overall survival based solely on clinical variables model. Diagonal dotted lines in the figure is the reference line, the predicted probability is equal to the probability of the actual situation, the farther off diagonal suggests that the bigger the error of the prediction. (1) If the predicted value is equal to the actual value, the plotted fit line coincides with the reference line. (2) If the predicted value is greater than the actual value, that is, the risk is overestimated, the fitted line is below the reference lin. (3) If the predicted value is less than the actual value, that is, the risk is underestimated, the fitted line is above the reference line

To verify the importance of PSAT1 expression in late-stage patients, we also developed prediction models based on other statistically significant clinical parameters (Fig. S2), except PSAT1. The AUCs were 0.696 for 3-year OS and 0.684 for 5-year OS (Fig. 4D-E). The results demonstrated that the predictive accuracy of the new model was significantly higher than that of model considering only clinical parameters. Further, the calibration plots of the new model suggested good agreement between the observed outcome and predicted probability unlike clinical parameter model (Fig. 5).

### Validation the expression of PSAT1 at different molecular levels

**Fig. 6:**
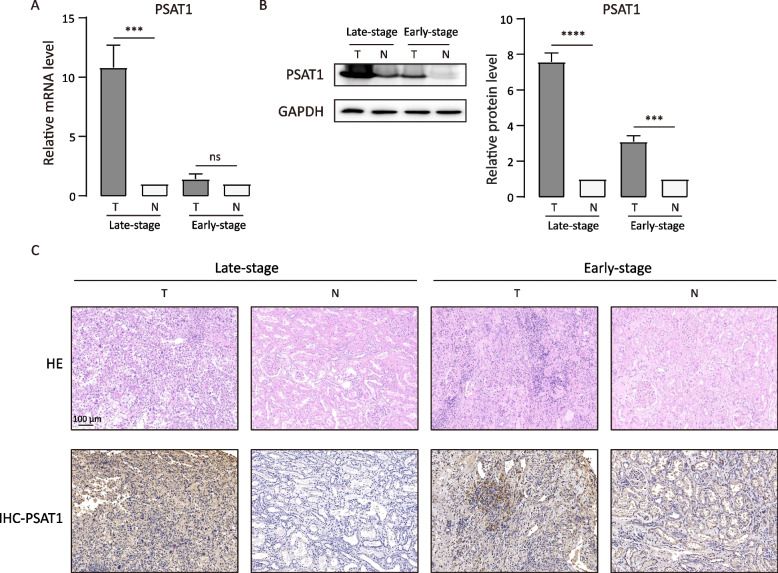
Verification of PSAT1 expression levels. **A** PSAT1 mRNA levels are elevated in 20 pairs of ccRCC tissues compared with matched tissues. **B** PSAT1 protein levels in 20 pairs of ccRCC tissues and matched noncancerous tissues were detected by WB. 20 pairs ccRCC samples: (10 pairs: late-stage; 10 pairs: early-stage). The abscissa represents the sample type, and the ordinate represents the relative expression values of mRNA or protein levels in tumor tissues compared with adjacent normal tissues. The level of the columns represents the level of expression. qRT-PCR and WB data are presented as the mean ± SD. (N: noncancerous, T: tumor; ****P < 0.0001, ***P < 0.001; ns: no significance). **C** The representative images of HE staining and PSAT1 immunohistochemistry in late-stage and early-stage ccRCC pair tissues. The scale bars indicate 100 µm

To validate the role of PSAT1 in ccRCC development and progression, we first examined PSAT1 expression status in ccRCC tissues. PSAT1 mRNA levels were detected in 20 ccRCC tissues and matched noncancerous adjacent tissues. The results demonstrated that the mRNA expression of PSAT1 was significantly increased in late-stage ccRCC tumor tissues (Fig. 6A).Quantitative values for each specific pair of tissues are described in Fig. S5A.

We further detected the protein expression levels of PSAT1 in 20 pairs of tumor and adjacent noncancerous tissues, including tissues from 10 pairs from early-stage and 10 pairs from late-stage cases. We observed that the PSAT1 expression was up-regulated in all tumor tissues compared with matched noncancerous tissues, and that the expression was higher in late-stage tumor tissues (Fig. 6B). Hematoxylin-Eosin (HE) staining showed that the tumor tissue had clear cell outline, transparent cytoplasm, centered nucleus, cells arranged in sheets, and less interstitium compared with the normal adjacent tissue, and the results were more obvious in patients with late-stage ccRCC (Fig. 6C). Immunohistochemical staining in serial sections also confirmed a positive correlation between the expression levels of PSAT1 in human ccRCC tumor tissues and the disease stage (Fig. 6C). The expression level was also higher in late-stage cases. In Fig. S5B, immunohistochemical scores and quantitative values of the proportion of positive cells in pathologically stained sections are described.

## Discussion

The prognosis of ccRCC patients is closely associated with tumor stage, with the later stages often indicating a poor prognosis [21]. Treatment options for advanced stage are limited. Currently, there is a scarcity of effective therapeutic strategies for recurrent and metastatic ccRCC [22]. Thus, the development of a new prognostic tool is crucial for identifying high-risk patients requiring additional treatment and attention. Moreover, finding a promising therapeutic target is crucial for developing anti-tumor drugs and improving the survival rate of patients with advanced ccRCC.

With the advancement of bioinformatics, an increasing number of genes have been identified as closely associated with ccRCC occurrence and development [23, 24]. Accordingly, we focused on gene expression in different stages of ccRCC using the GEO dataset and verified our findings with ACBI-TCGA. In our study, we found three DEGs (PSAT1, PRAME, and KDELR3) between late-stage patients and early-stage patients across three mRNA arrays. The post-match data included complete variables for comparison in both stage groups. Except for the stage parameter, another principal clinical factor for ccRCC prognosis is the grade parameter [25, 26]. We concluded that late-stage group patients also tended to have a higher disease grade. This further confirms that tumor staging and histological grading are the main parameters associated with ccRCC prognosis [27].

In survival analysis, we found that patients in the late stage of the disease showed a poor prognosis, and PSAT1 was the only gene associated with OS in this group. However, the relative expression of the target genes may be involved in the prognosis [23]. The ccRCC clinicopathological information downloaded from TCGA was processed via univariate and multivariate Cox regression analysis. Age, PSAT1 expression, grade, and neoadjuvant therapy were found to be significant independent prognostic factors associated with OS. Neoadjuvant therapy has been proved to be a protective factor. However, in previous studies, the benefit of neoadjuvant therapy for locally advanced ccRCC with currently available therapeutic agents has been controversial [28]. Christopher et al. [29] suggested that neoadjuvant treatment with pazopanib is effective in treating patients with localized ccRCC, which is similar to the findings of our study.

Various nomograms have been identified to determine the prognosis of ccRCC patients. For instance, Xia et al. [12] constructed a prognostic nomogram based on the prognostic risk signature and clinicopathological characteristics, which exhibited high accuracy and a robust predictive performance. The study by Zhu et al. [30] reported that combining methylation risk scores with conventional clinical covariates improved the prediction of clinical prognosis in ccRCC patients. In our study, we developed a new graphical nomogram that combines PSAT1 expression with clinicopathological data for predicting OS in ccRCC patients at different stages. By assigning values to clinical variables and PSAT1 expression for each patient, we calculated a total score that predicts the OS of late-stage patients at 3 and 5 years. The abovementioned estimates can be used for patient counseling and informed decision-making. The C-index, AUC, and calibration curve indicators from the entire TCGA set confirmed the discriminative accuracy of our nomogram, possibly making it a preferred predictive model. Moreover, the predictive power of our new model was higher than that of single clinical variable model.

Of the three hub-genes identified in our study, PSAT1 was finally included in the model to predict the survival of late-stage ccRCC patients. Previous research reported that the dysregulation of PSAT1 activity may alter glucose and glutamine utilization in serine biosynthesis, promoting tumorigenesis and chemoresistance in colorectal cancers given that PSAT1 is a metabolism-related gene [31, 32]. Increased transcription of PSAT1, caused by promoter hypomethylation, was also linked to a poor response to tamoxifen therapy and cancer recurrence in early-stage breast cancer [33, 34]. Furthermore, studies have shown that the up-regulation of PSAT1 promotes cell proliferation and is associated with a poor outcome in patients with non-small cell lung cancer [35, 36]. These studies indicate a strong correlation between PSAT1 levels and tumor progression as well as prognosis.

The confirmation of the association of PSAT1 expression levels with ccRCC indicates its involvement in metabolism, development, and progression. Zhang et al. [37] screened ccRCC-related glycolytic genes in public databases and constructed a prediction model of 13 genes including PSAT1, which could be valuable for diagnosing and predicting ccRCC. The study by Cheng et al. [38] introduced a new gene signature, including PSAT1, to determine the ccRCC prognosis in TCGA cohorts based on amino acid metabolism-related genes. In light of the GEPIA2 analysis, some other tumors (BLCA, CESC, COAD, DLBC, GBM, LGG, LUAD, LUSC, OV, PRAD, READ, STAD, THYM, UCEC, and UCS) are high expressed of PSAT1 (Fig. S4), which bodes well for the possibility of studying PSAT1 as a biomarker in other tumors. However, for advanced stage ccRCC patients, the role of PSAT1 remains elusive. In our research, PSAT1 was found to be highly expressed in patients with late-stage ccRCC and affected the OS of patients. Our model further demonstrated the application value of PSAT1 in accurately determining the prognosis in advanced ccRCC patients.

The results of mRNA and protein level validation may provide guidance for clinical decision making. For example, in clinical practice, when the clinical renal cell carcinoma patients after surgical treatment, the histopathology suggests that the renal cell carcinoma is clear cell carcinoma, the expression of PSAT1 can be further detected by immunohistochemistry. If PSAT1 expression is positive, the prognosis of the patients is poor, which provides a reference for clinicians for the next treatment of the patients.

To the best of our knowledge, this is the first nomogram to predict OS in patients with different stages of ccRCC by combining genetic information and clinical data. Furthermore, the significance of PSAT1 in late-stage ccRCC was confirmed in this study, providing more specific and precise insights on its role.

## Conclusion

The prognostic gene expression profiles of ccRCC patients at different stages were determined in this study. The high expression of PSAT1 in tumor tissues, especially late-stage tumor tissues, combined with clinical data, in this nomogram enhances its prognostic value in different-stage ccRCC patients, particularly for the prediction of OS.

### Supplementary Information

**Supplementary Material 1.**

**Supplementary Material 2.**

**Supplementary Material 3.**

**Supplementary Material 4.**

**Supplementary Material 5.**

## Data Availability

The datasets used and/or analyzed during the current study are available from the corresponding author on reasonable request.
