# Supplementary material for: PSAT1 enhances the efficacy of the prognosis estimation nomogram model in stage-based clear cell renal cell carcinoma
Source: BMC Cancer. 2024 Apr 13;24:463. doi: 10.1186/s12885-024-12183-z (PMC11016215; doi:10.1186/s12885-024-12183-z)
Supplement: Supplementary file 1 — Supplementary Material 1. [file 12885_2024_12183_MOESM1_ESM.pdf]

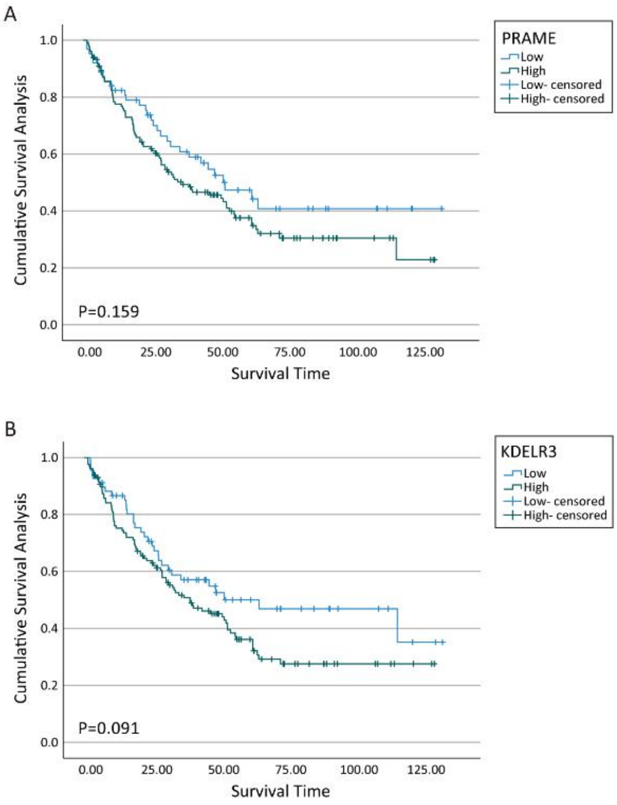

Supplement Figure 1

Supplementary Figure 1. Kaplan-Meier plots for KIRC patients.

(**A**) PRAME expression level about late-stage patients for overall survival. (**B**) KDELR3 expression level about late-stage patients for overall survival. The mortality rates of the different expression level showed a downward trend, which decreased rapidly at the beginning and then leveled off. The blue curve represents the high-expression, green curve represents the low-expression. Follow-up time for horizontal axis and vertical axis for survival rate. Survival curves were obtained by linking the corresponding survival rates at each time point. $p<0.05$ was considered statistically significant.
