# Supplementary material for: PSAT1 enhances the efficacy of the prognosis estimation nomogram model in stage-based clear cell renal cell carcinoma
Source: BMC Cancer. 2024 Apr 13;24:463. doi: 10.1186/s12885-024-12183-z (PMC11016215; doi:10.1186/s12885-024-12183-z)
Supplement: Supplementary file 2 — Supplementary Material 2. [file 12885_2024_12183_MOESM2_ESM.pdf]

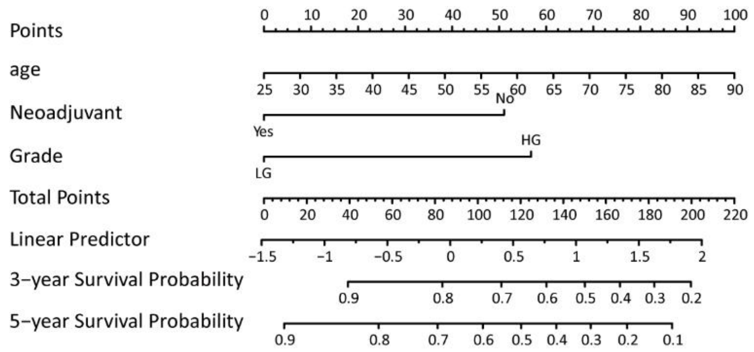

Supplement Figure 2

Supplementary Figure 2. Single clinical variables-based nomogram for predicting 3-year and 5-year overall survival. The risk factors were represented by points on the axis, with each factor corresponding to a line drawn upward. The total points located on the axis indicated the probability of 3-year and 5-year overall survival, represented by a line drawn downward to the survival axis.
