# Supplementary material for: PSAT1 enhances the efficacy of the prognosis estimation nomogram model in stage-based clear cell renal cell carcinoma
Source: BMC Cancer. 2024 Apr 13;24:463. doi: 10.1186/s12885-024-12183-z (PMC11016215; doi:10.1186/s12885-024-12183-z)
Supplement: Supplementary file 3 — Supplementary Material 3. [file 12885_2024_12183_MOESM3_ESM.pdf]

Uncropped scans of the Western blots shown in the indicated figures

Fig. 6b

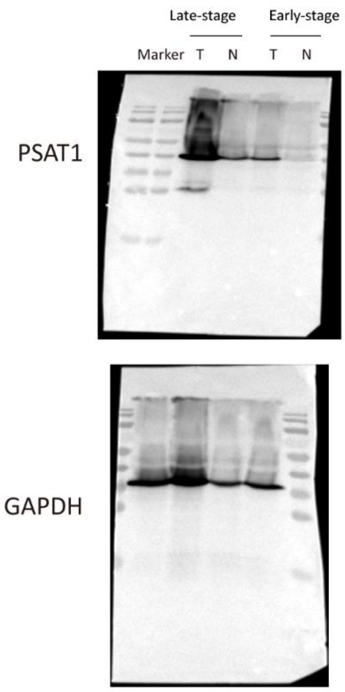

Supplement Figure 3

Supplementary Figure 3. Uncropped scans of the WB shown in the indicated figures.
