# Supplementary material for: PSAT1 enhances the efficacy of the prognosis estimation nomogram model in stage-based clear cell renal cell carcinoma
Source: BMC Cancer. 2024 Apr 13;24:463. doi: 10.1186/s12885-024-12183-z (PMC11016215; doi:10.1186/s12885-024-12183-z)
Supplement: Supplementary file 4 — Supplementary Material 4. [file 12885_2024_12183_MOESM4_ESM.pdf]

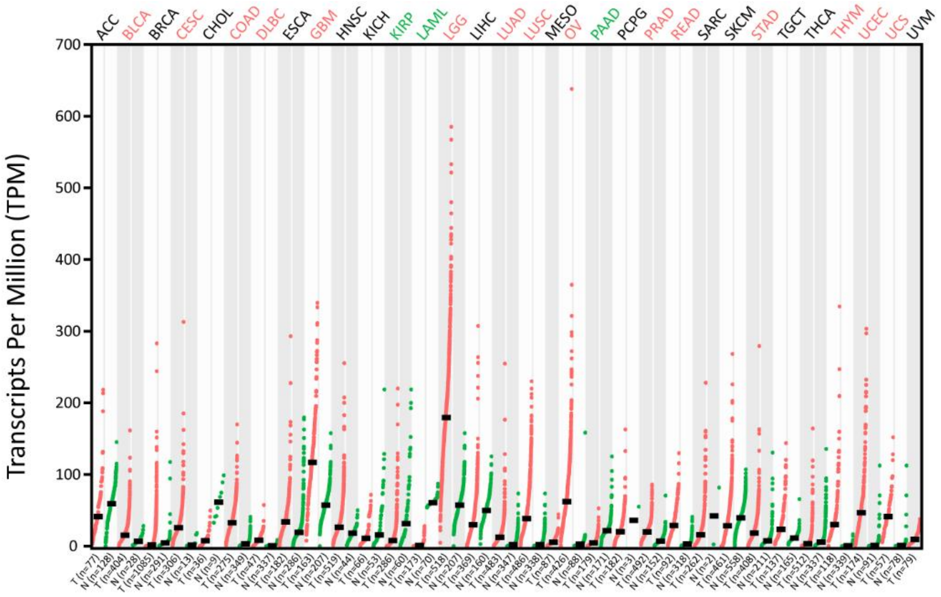

Supplement Figure 4

Supplementary Figure 4. The expression of PSAT1 in other tumor tissues.

Red represents high expression and green represents low expression with statistical difference. However, there was no statistically significant difference in PSAT1 expression in other tumors.

ACC: Adrenocortical carcinoma; BLCA: Bladder Urothelial Carcinoma; BRCA: Breast invasive carcinoma; CESC: Cervical squamous cell carcinoma and endocervical adenocarcinoma; CHOL: Cholangio carcinoma; COAD: Colon adenocarcinoma; DLBC: Lymphoid Neoplasm Diffuse Large B-cell Lymphoma; ESCA: Esophageal carcinoma; GBM: Glioblastoma multiforme; HNSC: Head and Neck squamous cell carcinoma; KICH: Kidney Chromophobe; KIRP: Kidney renal papillary cell carcinoma; LAML: Acute Myeloid Leukemia; LGG: Brain Lower Grade Glioma; LIHC: Liver hepatocellular carcinoma; LUAD: Lung adenocarcinoma; LUSC: Lung squamous cell carcinoma; MESO: Mesothelioma; OV: Ovarian serous cystadenocarcinoma; PAAD: Pancreatic adenocarcinoma; PCPG: Pheochromocytoma and Paraganglioma; PRAD: Prostate adenocarcinoma; READ: Rectum adenocarcinoma; SARC: Sarcoma; SKCM: Skin Cutaneous Melanoma; STAD: Stomach adenocarcinoma; TGCT: Testicular Germ Cell Tumors; THCA: Thyroid carcinoma; THYM: Thymoma; UCEC: Uterine Corpus Endometrial Carcinoma; UCS: Uterine Carcinosarcoma; UVM: Uveal Melanoma.
