# Supplementary material for: PSAT1 enhances the efficacy of the prognosis estimation nomogram model in stage-based clear cell renal cell carcinoma
Source: BMC Cancer. 2024 Apr 13;24:463. doi: 10.1186/s12885-024-12183-z (PMC11016215; doi:10.1186/s12885-024-12183-z)
Supplement: Supplementary file 5 — Supplementary Material 5. [file 12885_2024_12183_MOESM5_ESM.pdf]

A

Late-stage	mRNA level (related to matched noncancerous tissues)
Tumor 1	12.91
Tumor 2	10.26
Tumor 3	9.27
Tumor 4	13.90
Tumor 5	10.55
Tumor 6	12.82
Tumor 7	11.68
Tumor 8	10.83
Tumor 9	8.08
Tumor 10	7.78

Early-stage	mRNA level (related to matched noncancerous tissues)
Tumor 1	1.12
Tumor 2	1.33
Tumor 3	1.05
Tumor 4	1.76
Tumor 5	1.34
Tumor 6	1.88
Tumor 7	2.28
Tumor 8	0.96
Tumor 9	1.58
Tumor 10	0.77

B

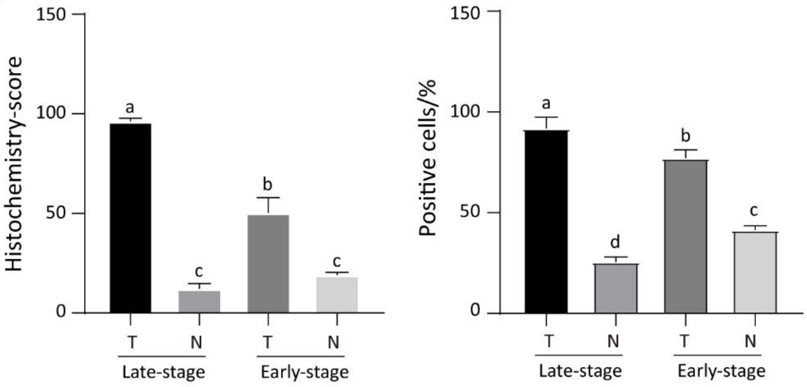

Supplement Figure 5

Supplementary Figure 5. Expression of PSAT1 at the ccRCC tissue level in 20 pairs of patients.

(A) Relative quantitative values of qRT-PCR for 20 pairs ccRCC tissue, which expression of each tumor tissue relative to noncancerous tissue.Left panel indicate late-stage, right panel indicate early-stage. **(B)** After IHC staining of 20 pairs of ccRCC tissues, the immunohistochemical score and positive cell rate of each pair of tissue sections were quantified. The quantitative histograms of renal tissue pathology are shown. Image-Pro Plus 6.0 was used to detect the parameters related to the number of positive cells in the immunohistochemical section and Histochemistry-score (H-score) was calculated. H-score is a value between 0 and 300, and the larger the value is, the stronger the comprehensive positive strength is in terms of the depth of positive and the number of positive.One-way ANOVA with the Tukey test was performed. $p<0.05$ was considered significant and indicated by different letters.
